# Risk-score performance for detecting transthyretin cardiac amyloidosis in severe aortic stenosis: a prospective cohort study

**DOI:** 10.3389/fcvm.2026.1773579

**Published:** 2026-05-22

**Authors:** Graczyk Katarzyna, Dziewięcka Ewa, Winiarczyk Mateusz, Stępień Agnieszka, Przytuła Natalia, Holcman Katarzyna, Wiśniowska-Śmiałek Sylwia, Szot Wojciech, Kostkiewicz Magdalena, Rubiś Paweł

**Affiliations:** 1 Doctoral School of Medical and Health Sciences, Jagiellonian University Medical College, Krakow, Poland; 2 Clinical Department of Cardiac and Vascular Diseases, John Paul II Hospital, Krakow, Poland; 3 Department of Nuclear Medicine, John Paul II Hospital, Kraków, Poland; 4 Department of Cardiac and Vascular Diseases, Institute of Cardiology, John Paul II Hospital, Jagiellonian University Medical College, Krakow, Poland

**Keywords:** amyloidosis, aortic stenosis, ATTR-CA, score, TAVI

## Abstract

**Introduction:**

The coexistence of transthyretin cardiac amyloidosis (ATTR-CA) and severe aortic stenosis (AS) presents a diagnostic challenge. This study aimed to determine the prevalence of this dual pathology and to assess the effectiveness of diagnostic models in enhancing detection accuracy.

**Methods:**

We conducted a prospective study of 104 consecutive patients with severe AS (aortic valve area <1 cm^2^). Comprehensive evaluation included clinical and laboratory assessments, electrocardiography, transthoracic echocardiography, planar whole-body bone scintigraphy, and chest single-photon emission computed tomography/computed tomography using technetium-99m-labelled 3,3-diphosphono-1,2-propanodicarboxylic acid tracer. Patients were grouped according to the presence of ATTR-CA, and the diagnostic utility of the RAISE score, its variations, and the T-AMYLO score was evaluated.

**Results:**

Nineteen (18%) patients with AS also had ATTR-CA (ATTR-CA-AS). They were significantly older (82.6 ± 7.4 vs. 76 ± 6.9, *p* < 0.001), more often had arrhythmic and conduction disorders (atrial fibrillation, implanted pacemakers), and had a reduced left ventricular ejection fraction (44.6% vs. 54.7%, *p* = 0.03). Patients with ATTR-CA-AS more frequently presented with the low-gradient/low-flow phenotype of AS (73.7%, *p* = 0.009). Both NT-proBNP and troponin levels were significantly higher in patients with ATTR-CA-AS (4174.0 vs. 1,238 pg/mL; *p* < 0.001; and 59.6 vs. 23.3 ng/L, *p* < 0.001, respectively). After a 6-month follow-up, a similar number of deaths occurred in both groups, and no surgical aortic valve replacement was performed in the ATTR-CA-AS population. The eRAISE model demonstrated the highest diagnostic accuracy (AUC=0.948).

**Conclusions:**

ATTR-CA is commonly observed in patients with severe AS. Implementing risk scores in routine practice could enhance the diagnostic accuracy of ATTR-CA in patients with AS, given their strong diagnostic performance and ease of assessment.

## 1 Introduction

Aortic stenosis (AS) is the most common primary valve lesion in developed countries and predominantly affects older adults, reflecting population ageing. Transthoracic echocardiography (TTE) is the method of choice for diagnosing AS and forms the basis for an optimal management strategy, including the selection of patients for intervention. However, identifying concomitant cardiac conditions that also manifest with left ventricular (LV) hypertrophy can be challenging, despite their clinical relevance in optimizing medical management before and after valve intervention. Amyloidosis, a systemic disease, has recently been identified as a coexisting condition in a substantial number of cases (1).

Amyloidosis is an infiltrative disease that varies according to the type of deposited protein. The two most common forms affecting the heart are light-chain amyloidosis (AL), resulting from the deposition of misfolded immunoglobulin light chains, and transthyretin amyloidosis (ATTR), caused by the accumulation of misfolded transthyretin, either due to a pathogenic gene variant (variant ATTR, vATTR) or, more commonly, due to age-related destabilization of the wild-type protein (wild-type ATTR, wtATTR). Cardiac involvement due to the course of amyloidosis is termed cardiac amyloidosis (CA). The reported incidence of ATTR varies from 6.1 per million in the United States to 232 per million in Portugal (2). In cases of heart failure with preserved ejection fraction (HFpEF), the incidence is estimated at 1.5% and increases with age, reaching 21% in patients aged 90 years and older (3). For years, ATTR-CA was largely of academic interest, as a definite diagnosis required an invasive endomyocardial biopsy. However, in recent years, the diagnostic pathway has changed substantially following Perugini's introduction of bone scintigraphy as a safer and more accessible test (4). Along with this simplified diagnostic approach, novel therapeutic agents have been developed and introduced worldwide. The identification of clinical indicators (“red flags”) is crucial for raising suspicion of ATTR-CA, and severe AS has recently been recognized as one such indicator. The coexistence of severe AS in patients with ATTR-CA is still considered to be relatively rare; therefore, screening every patient with AS for concomitant ATTR is neither feasible nor economically viable (5). Given this fact, a credible screening tool is needed to accurately identify patients with concomitant ATTR-CA and AS. Therefore, implementing a validated and easy-to-use diagnostic score may be of key importance.

## 2 Objectives

This prospective cohort study aimed to (1) determine the prevalence and clinical characteristics of concomitant ATTR-CA among consecutive patients with severe aortic stenosis in southeast Poland and (2) externally validate existing diagnostic risk scores/models for ATTR-CA in patients with severe AS.

## 3 Methods

### 3.1 Study population and protocol

We prospectively enrolled consecutive patients with severe AS aged ≥65 years who were managed at our tertiary hospital, including urgent and elective admissions and outpatients, from May 2023 to August 2025, with no contraindication or limitation to SPECT/CT (including myocardial infarction within the last 6 weeks, hydroxychloroquine treatment, general frailty or inability to lie supine for the duration of the scan, or significant renal insufficiency) (6). All patients underwent planar whole-body bone scintigraphy, and, in case of positive results, accompanying chest single-photon emission computed tomography/computed tomography (SPECT/CT) with technetium-99m-labelled DPD [(99mTc)-DPD], as well as clinical and laboratory assessment, electrocardiography (ECG), and TTE. During the index visit, all patients underwent a standardized assessment that included a medical history focused on established non-cardiac ATTR “red flags,” such as carpal tunnel syndrome, lumbar stenosis, ruptured biceps tendon, and peripheral polyneuropathy (7). Physical examination focusing on signs of congestion was also performed. In addition, a review of the current pharmacotherapy was conducted.

Written informed consent was obtained from all enrolled participants. All procedures were carried out in accordance with the ethical principles approved by the Jagiellonian University Bioethics Committee (1072.6120.37.2023), the 1964 Helsinki Declaration, and its subsequent amendments, or equivalent ethical standards (8).

### 3.2 ECG protocol

A resting 12-lead ECG was conducted according to the guidelines. Low limb lead voltages were defined as an amplitude of 0.5 mV in all limb leads and/or ≤1.0 mV in precordial leads (9).

Laboratory tests included blood and urine protein immunofixation, serum-free light-chain (sFLC) quantification (Freelite® f-my, The Binding Site Group Ltd., UK), N-terminal pro-brain natriuretic peptide (NT-proBNP), high-sensitivity troponin T (hs-TnT), serum creatinine, lipid profile, and blood count.

### 3.3 Echocardiographic assessment

Comprehensive transthoracic echocardiographic examinations were performed by specialized cardiologists (M.W. and K.G.) using a commercially available ultrasound system (EPIQ 7 Cardiology Ultrasound Machine, Philips) with a 3.5-MHz transducer. All parameters were highly reproducible at both the intra- and interobserver levels. Bland–Altman analysis showed the narrowest limits of agreement (Supplementary Figure S4). The 95% limits of agreement for each parameter are reported in the corresponding table. In the presence of atrial fibrillation (AF), the measurements were averaged over five cardiac cycles; otherwise, three cycles were obtained. LV end-systolic and end-diastolic volumes, along with the LV ejection fraction (LVEF), were measured using the biplane Simpson approach. The left ventricular mass and its index were calculated according to the Devereux formula (10) (Supplementary Appendix S1).

Severe AS was diagnosed through echocardiography according to the current ESC guidelines when the estimated aortic valve area (AVA) was <1.0 cm^2^ (1). The following AS categories were considered: (1) high-gradient (HG) (mean aortic gradient ≥40 mmHg); (2) low-flow, low-gradient (LF/LG) with reduced ejection fraction [mean aortic gradient <40 mmHg, LVEF<50%, and stroke volume index(Svi) ≤35 mL/m^2^]; (3) LF/LG with preserved LVEF (mean aortic gradient <40 mmHg, LVEF≥50%, and SVi ≤35 mL/m^2^); and (4) normal-flow, low-gradient (NF/LG) AS with preserved EF (mean gradient <40 mmHg, AVA ≤1 cm^2^, SVi >35 mL/m^2^, and LVEF ≥50%) (11) (Supplementary Appendix S1).

### 3.4 SPECT/DPD protocol

Whole-body planar and chest SPECT images were acquired at rest in the supine position 2–3 h after intravenous injection of technetium-99 m (370–740 MBq) and DPD tracers (TECEOS and CIS BIO), in accordance with current guidelines. Images were reviewed in standard cardiac imaging planes using commercial software by certified nuclear medicine specialists (M.K., W.S., and K.H.) according to the semi-quantitative scale proposed by Perugini et al.: grade 0—no myocardial uptake with normal bone uptake; grade 1—myocardial uptake less than rib uptake; grade 2—myocardial uptake equal to rib uptake; and grade 3—myocardial uptake greater than rib uptake with mild/absent rib uptake (Supplementary Figure S1). Both CT attenuation-corrected and non-corrected SPECT images were evaluated by experienced nuclear medicine specialists. The assessment included coronal, transaxial, and sagittal views, as well as a three-dimensional maximum-intensity projection cine mode to provide a comprehensive analysis of the imaging data (12).

### 3.5 ATTR-CA diagnosis

Perugini grading was used in the diagnostic path for qualitative assessment (0, negative; 1, inconclusive; 2–3, positive). ATTR-CA was diagnosed in patients with Perugini grade 2–3 on DPD scintigraphy in the absence of clonal dyscrasia, assessed by sFLC assay, serum and urine protein electrophoresis, and immunofixation to detect pathological light chains or a monoclonal component. In the presence of monoclonal gammopathy, patients underwent bone marrow biopsy at a referral centre. The diagnostic algorithm proposed by Gillmore et al. and endorsed by the ESC has a specificity and positive predictive value for ATTR-CA diagnosis of almost 100% (98.0–100; Supplementary Figure S2) (13). Patients diagnosed with AL-CA were excluded from further statistical analyses (Supplementary Figure S3).

Genetic analysis of the TTR gene was conducted following a positive result on bone scintigraphy. This study used a next-generation sequencing approach to assess the TTR gene. The TTR gene was analyzed in a certified diagnostic laboratory using amplicon-based next-generation sequencing, covering the entire coding region and conserved splice junctions (14).

### 3.6 ATTR diagnostic models

The following models for estimating the probability of an ATTR-CA diagnosis were computed: (1) RAISE (Remodeling, Age, Injury, System, and Electrical) models, including (a) the original (oRAISE), (b) modified (mRAISE), and (c) enriched (eRAISE) versions; and (2) T-AMYLO (Tunnel, Age, Male, hYpertrophy, LOw voltage) simplified score (for details, see Supplementary Appendix S1) (15–17). The RAISE score is intended for patients with severe AS who are being considered for TAVI (transcatheter valve implantation). It combines the following factors, each assigned a certain number of points: clinical—age ≥85 years; history of carpal tunnel syndrome; laboratory—high-sensitivity troponin T (hs-TnT) >20 ng/L; ECG—right bundle branch block (RBBB); low voltages or Sokolow–Lyon index <1.9 mV; echocardiography—marked LV hypertrophy (septal wall thickness ≥18 mm); and diastolic dysfunction (E/A ratio >1.4).

T-AMYLO includes different patient phenotypes (HFpEF, AS, acute HF) and uses a combination of readily available clinical information, ECG findings, and echocardiographic measurements to estimate a patient's risk. The final score was composed of age ≥80 years, male gender, carpal tunnel syndrome, IVSd ≥16 mm, low QRS voltage, and RBBB.

### 3.7 Follow-up

After 6 months, a telephone follow-up was conducted, along with a review of hospital medical records, focusing on survival and the performance of valvular intervention.

### 3.8 Statistical analysis

All parameters are presented as mean ± standard deviation, median (interquartile range), or count (percentage). The normality of the quantitative variables was assessed using the Shapiro–Wilk test. Comparisons between groups were performed using the *t*-test, Mann–Whitney U test, *χ*^2^ test, or Fisher's exact test, as appropriate. A univariable regression model was conducted using baseline parameters as covariates, with the presence of ATTR-CA serving as the response variable. Spearman’s correlation was used for the internal correlation assessment. The four analysed scores (oRAISE, mRAISE, eRAISE, and T-AMYLO) were evaluated, and the areas under the receiver operating characteristic (ROC), referred to as the area under the curve (AUC), were calculated to assess their discriminative ability. Cut-off points were proposed based on the sensitivity and specificity. The loop diuretic dosage was calculated as the sum of the daily dose of furosemide and three times the daily dose of torsemide. Statistical significance was defined as a *p*-value <0.05. All statistical analyses were performed using the Statistica package, version 13 (StatSoft, TIBCO Software Inc.).

## 4 Results

### 4.1 ATTR-CA in patients with severe AS

A total of 104 patients with AS (mean age, 77.6 ± 6.9 years; 41% female) were analysed. Overall, 24 (22.3%) patients were diagnosed with CA, including 19 (18.3%) with ATTR-CA and 5 (4.8%) with AL-CA. Among the patients diagnosed with ATTR-CA, all demonstrated high Perugini grades, including grade 2 (*n* = 3, 16%) and grade 3 (*n* = 16, 84%) uptake on DPD scintigraphy (Supplementary Table S2). Patients suspected of AL-CA (mean age 78.8 years; 40% female; LVEF 46.2%; NT-proBNP 7,205.6 pg/dL) were referred to haematology specialists and were excluded from further analysis. The remaining study population consisted of patients with ATTR-CA and severe AS (ATTR-CA-AS; *n* = 19) and those with severe AS alone (*n* = 80). Patients with ATTR-CA-AS were significantly older than those with severe AS alone, and no difference in sex distribution was noted. They more frequently exhibited symptoms of congestion. Among the symptoms identified as red flags for ATTR, a history of carpal tunnel syndrome was more common in patients with ATTR-CA-AS compared to those with severe AS alone [8 (42.1%) vs. 3 (3.7%), *p* < 0.001]. Another red flag, lumbar canal stenosis, also occurred significantly more often with a dual diagnosis [3 (15.8%) vs. 1 (1.2%), *p* = 0.005]. Peripheral polyneuropathy and ruptured biceps tendons are uncommon. With regard to comorbidities, both cohorts had a similar percentage of coronary artery disease and type 2 diabetes mellitus (T2DM); however, AF was more frequent in the ATTR-CA-AS group [13 (68.4%) vs. 29 (36.2%), *p* = 0.011]. The incidence of pulmonary embolism was also higher in the ATTR-CA-AS group [3 (15.8%) vs. 3 (3.7%), *p* = 0.053]. Patients with ATTR-CA-AS more often developed arrhythmias, including ventricular extrasystoles and conduction disorders, resulting in more frequent pacing device implantation [8 (42.1%) vs. 7 (8.7%), *p* < 0.001]. However, characteristic ECG findings, including low QRS voltage and pseudo-infarct patterns, did not differ markedly between the groups.

On TTE, concomitant AS and ATTR-CA were associated with greater interventricular septal and posterior wall thickness and a higher LV mass index (LVMI 187 ± 71.6 g/m^2^ vs. 133.3 ± 40.3 g/m^2^, *p* = 0.001), consistent with more pronounced hypertrophy. Enlargement of both atria and increased right ventricular basal dimensions reflected more advanced diastolic dysfunction, as indicated by a higher E/E′ ratio. Globally, patients with ATTR-CA-AS exhibited worse left and right ventricular function, characterized by a lower LVEF (44.6 ± 17 vs. 54.7 ± 13.9, *p* = 0.026), more severely impaired global longitudinal strain (GLS), and lower TAPSE (17.8 ± 5.9 vs. 21.1 ± 5.4, *p* = 0.041).

In addition, patients with concomitant ATTR-CA had a significantly lower mean aortic gradient (25.4 ± 19.4mmHg vs. 38.1 ± 16.2, *p* = 0.02) and lower indexed stroke volume (22.9 ± 12.4 mL/m^2^ vs. 30.1 ± 17.3 mL/m^2^, *p* = 0.023), more frequently manifesting a low-flow/low-gradient (LF/LG) AS phenotype [16 (84.2%) vs. 36 (45%)], predominantly in association with reduced LVEF. Laboratory data were consistent with more advanced disease, with higher NT-proBNP [4,174 (1,767–10,881) vs. 1,238 (402–3,419), *p* < 0.001] and hs-TnT [54 (30–75) vs. 22 (14–31), *p* < 0.001] levels. Renal function was also more impaired, with higher serum creatinine levels and lower eGFR.

Patients with ATTR-CA-AS frequently present with congestion and require higher doses of loop diuretics. Nevertheless, general pharmacotherapy did not differ between the groups, indicating good tolerance of guideline-directed HF therapy. All patients with ATTR-CA-AS had negative genetic test results for the TTR gene, confirming a diagnosis of wtATTR-CA.

### 4.2 Follow-up

Patients with severe AS and concomitant ATTR-CA have a greater risk of valvular intervention. EuroSCORE II was significantly higher among patients with amyloidosis, and as a result, all were qualified for TAVI, balloon aortic valvuloplasty, or conservative treatment (often due to prohibitive procedural risk). During the first 6 months of observation, six patients with ATTR-CA-AS (31.6%) underwent successful TAVI. As for complications, pacemaker implantation after TAVI occurred more frequently in patients with ATTR-CA-AS (4, 66.7%) compared to those with isolated AS (4, 15.4%; *p* = 0.003). Surgical aortic valve replacement was performed only in patients with AS without ATTR-CA [18 (22.5%), *p* = 0.022]. Overall, 10 patients died, with a non-significant increase in the ATTR-CA-AS group. Deaths in the non-ATTR group occurred mainly shortly after the surgery. Moreover, 15 (78.9%) patients with AS-ATTR-CA were started on disease-modifying therapy with tafamidis during follow-up, while the remaining two patients died during the qualification process, and two patients were considered to be already in too advanced a state for implementing treatment with tafamidis (NYHA class III/IV and advanced multiorgan failure) (Table 1).

**Table 1:** Baseline clinical characteristics of patients with severe aortic stenosis, stratified by the presence of concomitant transthyretin cardiac amyloidosis (ATTR-CA).

Variable	All *N* = 99	With ATTR-CA, *N* = 19 (19.19%)	Without ATTR-CA, *N* = 80 (80.81%)	*p*-value
Sex (women)	41 (41.4%)	5 (26.3%)	36 (45.0%)	0.196
Age, years	77.42 ± 6.82	82.63 ± 3.39	76.04 ± 6.94	<0.001
BMI, kg/m^2^	28.3 ± 4.97	26.64 ± 3.52	28.7 ± 5.12	0.094
BSA, m^2^	1.86 ± 0,19	1.87 ± 0.14	1.86 ± 0.2	0.881
Congestion, *n* (%)	43 (40.9%)	13 (68.4%)	25 (33.3%)	0.003
NYHA				
I, *n* (%)	13 (13.1%)	1 (5.3%)	12 (15%)	*P* = 0.791
II, *n* (%)	43 (43.4%)	9 (47.4%)	34 (42.5%)	
III/IV, *n* (%)	43 (43.4%)	9 (47.4%)	34 (42.5%)	
EuroSCORE II	3.86 ± 3.30	5.51 ± 3.83	3.46 ± 3.04	0.002
Non-cardiac ATTR red flags				
Peripheral polyneuropathy, *n* (%)	4 (4.04%)	1 (5.26%)	3 (3.75%)	0.781
Lumbar canal stenosis, *n* (%)	4 (4.04%)	3 (15.79%)	1 (1.25%)	0.005
Ruptured biceps tendon, *n* (%)	0 (0%)	0 (0%)	0 (0%)	0
Carpal tunnel syndrome, *n* (%)	11 (11.11%)	8 (42.1%)	3 (3.75%)	<0.001
Comorbidities				
MGUS, *n* (%)	12 (12.12%)	5 (26.32%)	7 (8.75%)	0.1
T2DM, *n* (%)	41 (41.41%)	10 (52.63%)	31 (38.75%)	0.329
Coronary artery disease, *n* (%)	40 (33.65%)	7 (36.84%)	33 (38.37%)	0.085
AF/AT/Afl, *n* (%)	47 (47.47%)	13 (68.4%)	29 (36.25%)	0.011
PE, *n* (%)	6 (6.06%)	3 (15.79%)	3 (3.75%)	0.053
Electrophysiologic characteristics				
HR	72.76 ± 14.02	74.74 ± 10.6	70.68 ± 14.74	0.287
PR, ms	162.46 ± 35.4	180 ± 42.76	160.7 ± 33.96	0.268
QRS, ms	100.1 ± 30.75	125.26 ± 33.89	92.47 ± 26.82	<0.001
LBBB, *n* (%)	8 (8.1%)	3 (15.78%)	5 (6.25%)	0.353
RBBB, *n* (%)	9 (9.01%)	4 (21.05%)	5 (6.25%)	0.081
Pseudo-infarct pattern, *n* (%)	9 (9.01%)	4 (21.05%)	5 (6.25%)	0.06
Low QRS voltage, *n* (%)	9 (9.01%)	3 (15.79%)	6 (7.5%)	0.312
Ventricular arrhythmia, *n* (%)	18 (18.18%)	9 (47.37%)	9 (11.25%)	<0.001
Pacemaker, *n* (%)	15 (15.15%)	8 (42.11%)	7 (8.75%)	<0.001
ICD, *n* (%)	4 (4.04%)	1 (5.26%)	3 (3.53%)	0.73
CRT, *n* (%)	3 (3.03%)	2 (10.53%)	1 (1.18%)	0.035
DDD/VVI, *n* (%)	12 (12.12%)	7 (36.84%)	5 (6.25%)	<0.001
Echocardiographic characteristics				
LVEF, %	52.8 ± 14.96	44.58 ± 17.02	54.68 ± 13.92	0.026
IVSd, mm	14.22 ± 3.16	17.05 ± 3.85	13.51 ± 2.58	<0.001
LVEDD, mm	47.97 ± 8.18	47 ± 6.95	48.37 ± 8.56	0.664
PWd, mm	12.78 ± 2.82	15.76 ± 3.36	12.09 ± 2.15	<0.001
EDV, mL	121.07 ± 58.4	115.63 ± 48.29	122.54 ± 59.94	0.980
LVMI, g/m^2^	143.53 ± 52.48	187 ± 71.59	133.27 ± 40.29	0.001
LVM, g	267.42 ± 100.93	349.68 ± 132.43	249.03 ± 79.72	0.001
LAV, mL	119.55 ± 40.57	131.27 ± 42.84	116.59 ± 41.5	0.365
LAVi, mL/m^2^	58.22 ± 19.38	66.60 ± 20.27	56.42 ± 18.41	0.043
RAA, cm^2^	23.03 ± 6.93	29.33 ± 6.09	21.49 ± 7.23	<0.001
RVD1, mm	41.17 ± 6.26	46.79 ± 5.18	39.79 ± 5.8	<0.001
TAPSE, mm	20.46 ± 5.54	17.84 ± 5.92	21.07 ± 5.4	0.041
RVSP, mmHg	30.78 ± 11.65	31.22 ± 8.45	30.79 ± 12.72	0.588
RV S, cm/s	10.25 ± 2.91	8.63 ± 2.22	10.89 ± 2.9	0.010
RV wall thickness, mm	7.06 ± 1.58	7.52 ± 1.75	6.7 ± 1.33	0.141
LASR, %	15.72 ± 10.59	11.32 ± 6.94	16.53 ± 11.39	0.123
GLS, %	13.22 ± 4.62	11.07 ± 4.84	13.99 ± 4.4	0.035
E/E'	14.75 ± 5.21	16.98 ± 4.82	14.29 ± 6.46	0.037
Asc aorta, mm	36.31 ± 5.06	37 ± 3.96	35.85 ± 5.28	0.525
LVOT diameter, mm	21.03 ± 2.11	21.06 ± 1.95	20.99 ± 2.15	0.781
E/A	1.06 ± 0.82	1.76 ± 1.48	0.96 ± 0.62	0.049
VCI, mm	19.48 ± 5.12	22.13 ± 7.01	18.81 ± 4.47	0.123
pericardial effusion, *n* (%)	26 (25%)	6 (31.58%)	20 (23.53%)	0.384
Categories of aortic stenosis				
SVI, mL/m^2^	28.86 ± 16.90	22.94 ± 12.38	30.32 ± 17.26	0.023
SV (VTI), mL	65.0 ± 21.2	50.94 ± 14.92	68.77 ± 21.44	0.003
LVOT VTI, cm	19.08 ± 5.48	16.03 ± 3.97	19.92 ± 5.69	0.035
AV maximal gradient, mmHg	61.21 ± 27.61	44.89 ± 33.14	65.45 ± 25.2	0.005
AV mean gradient, mmHg	36.42 ± 17.46	25.37 ± 19.41	39.26 ± 16.23	0.002
LFLG	52 (52.5%)	16 (84.2%)	36 (45.0%)	0.002
With preserved LVEF	33 (33.3%)	6 (31.6%)	27 (33.8%)	0.708
With reduced LVEF	19 (19.2%)	10 (52.6%)	9 (11.3%)	<0.001
NFLG	11 (10.2%)	2 (10.5%)	9 (10.5%)	1.0
Laboratory parameters				
NT-proBNP, pg/mL	1,542.5 (–)	4,174.0 (1,767–10,881)	1,238 (402.5–3,419.75)	<0.001
hs-TnT, ng/dL	32.85 ± 29.35	59.58 ± 43.46	23.29 ± 24.09	<0.001
		54 (30–75)	22 (14–31.5)	
Creatinine, mg/dL	1.08 ± 0.58	1.35 ± 0.53	1.01 ± 0.58	0.006
eGFR, mL/min/1.73 m^2^	62.15 ± 20.19	51.53 ± 19.55	64.85 ± 20.41	0.021
Non-HDL cholesterol, mmol/L	2.77 ± 1.07	2.55 ± 0.87	2.83 ± 1.09	0.541
LDL cholesterol, mmol/L	2.48 ± 1.10	2.25 ± 0.89	2.54 ± 1.12	0.517
WBC, 10^3^	7.18 ± 2.5	6.86 ± 1.61	7.27 ± 2.64	0.618
PLT, 10^3^	200.93 ± 80.52	203.95 ± 69.22	200.37 ± 84.27	0.769
Hb, g/dL	13.07 ± 1.67	12.9 ± 1.74	13.16 ± 1.72	0.551
Hct, %	38.86 ± 4.64	38.78 ± 4.82	39.04 ± 4.91	0.825
TTR sequencing, *n* (%) pathogenic	0 (0.0%)	0 (0.0%)		
Pharmacotherapy				
BB, *n* (%)	83 (83.83%)	16 (84.21%)	67 (85.88%)	0.851
Amiodarone, *n* (%)	4 (4.04%)	1 (5.26%)	3 (3.75%)	0.789
MRA, *n* (%)	47 (47.47%)	11 (57.89%)	36 (44.71%)	0.358
ACEI, *n* (%)	67 (67.67%)	10 (52.63%)	57 (71.76%)	0.069
ARNI, *n* (%)	6 (6.06%)	1 (5.26%)	5 (6.25%)	0.843
SGLT2i, *n* (%)	52 (52.52%)	12 (63.16%)	40 (51.76%)	0.38
Loop diuretics, mg/day	20.06 ± 39.22	80 (15–350)	15 (0–37.5)	0.009
DOAC, *n* (%)	44 (44.44%)	12 (63.16%)	27 (33.75%)	0.023
Vitamin K antagonist, *n* (%)	5 (5.05%)	1 (5.26%)	4 (5.0%)	0.98
Tafamidis, *n* (%)		15 (78.9%)		
Models				
oRISE	1.83 ± 1.79	4.16 ± 1.8	1.28 ± 1.25	<0.001
mRISE	1.3 ± 1.56	3.37 ± 1.6	0.81 ± 1.07	<0.001
eRISE	0.12 ± 3.09	4.16 ± 1.68	−0.84 ± 2.5	<0.001
T-AMYLO	3.05 ± 2.46	5.74 ± 2.51	2.41 ± 1.97	<0.001
Follow-up				
Valve intervention, *n* (%)	53 (53.53%)	7 (36.84%)	46 (57.5%)	0.146
TAVI, *n* (%)	32 (32.32%)	6 (31.58%)	26 (32.5%)	0.938
Periprocedural TAVI complications				
Permanent pacemaker implantation, *n* (%)	8 (25%)	4 (66.7%)	4 (15.4%)	0.003
Infection, *n* (%)	6 (18.8%)	1 (16.7%)	5 (19.2%)	0.885
Moderate/severe paravalvular leak, *n* (%)	1 (3.1%)	0	1 (3.8%)	0.625
SAVR, *n* (%)	18 (18.18%)	0 (0%)	18 (22.5%)	0.022
BAV, *n* (%)	3 (3.03%)	1 (5.26%)	2 (2.5%)	0.528
Death, *n* (%)	10 (10.0%)	4 (21.1%)	6 (7.5%)	0.005
Periprocedural	4	0	4	
Without procedure	6	4	2	

ACEi, angiotensin-converting enzyme inhibitor; AF, atrial fibrillation; Afl, atrial flutter; ARNI, angiotensin receptor–neprilysin inhibitor; AT, atrial tachycardia; AV, aortic valve; BAV, balloon aortic valvuloplasty; BB, beta-blockers; BMI, body mass index; BSA, body surface area; CRT, cardiac resynchronization therapy; DOAC, direct oral anticoagulant; EDV, end-diastolic volume; eGFR, estimated glomerular filtration rate; EuroScore II, European System for Cardiac Operative Risk Evaluation II; GLS, global longitudinal strain; Hb, haemoglobin; Hct, haematocrit; HR, heart rate; hs-TNT, high-sensitive troponin T; ICD, implantable cardioverter-defibrillator; IVSd, intraventricular septum diastolic diameter; LAVI, left atrial volume indexed; LBBB, left bundle branch block; LDL, low-density lipoprotein; LFLG, low-ﬂow; low-gradient; LVEDd, left ventricle end-diastolic diameter; LVEF, left ventricle ejection fraction; LVMI, left ventricular mass indexed; LVOT, left ventricular outflow tract; LVOT VTI, left ventricular outflow tract velocity-time integral; MGUS, monoclonal gammopathy of undetermined significance; MRA, mineralocorticoid receptor antagonist; NFLG, normal-flow, low-gradient; non-HDL, non-high-density lipoprotein; NT-proBNP, N-terminal prohormone of brain natriuretic peptide; NYHA, New York Heart Association class; PE, pulmonary embolism; PLT, platelets; PWd, posterior wall diastolic diameter; RAA, right atria area; RBBB, right bundle branch block; RV, right ventricle; RVD1, right ventricle basal dimension; RVSP, right ventricular systolic pressure; SAVR, surgical aortic valve replacement; SGLT2is, sodium–glucose cotransporter 2 inhibitors; SVI, stroke volume indexed; T2DM, Type 2 diabetes mellitus; TAPSE, tricuspid annular plane systolic excursion; TAVI, transcatheter aortic valve implantation; TTR, transthyretin; WBC, white blood cells.

### 4.3 Identification of ATTR-CA among patients with AS

A univariable regression analysis was conducted to identify potential predictors of ATTR-CA in patients with severe AS. Several clinical and echocardiographic parameters were significantly associated with ATTR-CA. Notably, older age was associated with this condition. Among the echocardiographic measurements, IVSd and LVMI were strongly associated. Systolic function of both ventricles (TAPSE and LVEF) was inversely associated, whereas QRS duration and hs-TnT levels were significantly associated with ATTR-CA, with higher values correlating with a greater likelihood of ATTR-CA. Creatinine levels showed a trend towards significance (Table 2).

**Table 2:** Univariable regression analyses to identify predictors of transthyretin cardiac amyloidosis (ATTR-CA) in patients with severe aortic stenosis.

Parameters	Univariable	*p*-value
	OR [95%CI]	
Age	1.16 [1.06–1.27]	0.001
BMI	0.91 [0.81–1.02]	0.010
LVEF	0.96 [0.93–0.99]	0.01
IVSd	1.4 [1.17–1.68]	<0.001
LVMI	1.02 [1.01–1.03]	<0.001
TAPSE	0.91 [0.82–1.00]	0.045
AV mean gradient	0.95 [0.92–0.99]	0.008
QRS	1.03 [1.01–1.05]	<0.001
hs-TnT	1.03 [1.01–1.05]	0.004
Creatinine	2.20 [0.92–5.29]	0.07

AV, aortic valve; BMI, body mass index; hs-TNT, high-sensitive troponin T; IVSd, intraventricular septum diastolic diameter; LVEF, left ventricle ejection fraction; LVMI, left ventricular mass indexed; TAPSE, tricuspid annular plane systolic.

Patients with ATTR-CA-AS had an average oRISE score of 4.16 ± 1.8, indicating a higher burden of disease compared to those without ATTR-CA, who had a significantly lower mean score of 1.28 ± 1.25. The average mRISE score for patients with ATTR-CA-AS was 3.37 ± 1.6 vs. 0.81 ± 1.07 in those without ATTR-CA. In terms of the eRISE score, patients with ATTR-CA-AS had a mean score of 4.16 ± 1.68, compared with a negative mean score of −0.84 ± 2.5 for those without ATTR-CA. The mean T-AMYLO score was markedly higher in patients with ATTR-CA-AS (5.74 ± 2.51) than in those without ATTR-CA (2.41 ± 1.97).

The eRAISE score demonstrated the best diagnostic performance for identifying ATTR-CA among patients with severe AS, with an AUC of 0.948 (95%CI 0.901–0.995) and high sensitivity and specificity at the cut-off point of 3 points proposed by the authors of the score (84.2% and 88.5%, respectively). The oRAISE model exhibited an AUC of 0.893 (95%CI 0.81–0.97), with a sensitivity and specificity of 78.9% and 76.5%, respectively, at a threshold of ≥3 points, and improved sensitivity (89.5%) with lower specificity (64.1%) at ≥2 points. A score of ≥4 points was indicated as a confirmatory marker, with a sensitivity of 68.4% and a high specificity of 92.3%. The mRAISE model demonstrated an AUC of 0.905 (95%CI 0.84–0.97). At a cut-off of ≥3 points, the sensitivity and specificity were 68.4% and 88.5%, respectively, while at ≥2 points, the sensitivity was 84.2% and the specificity was 80.8%.

**Figure 1:**
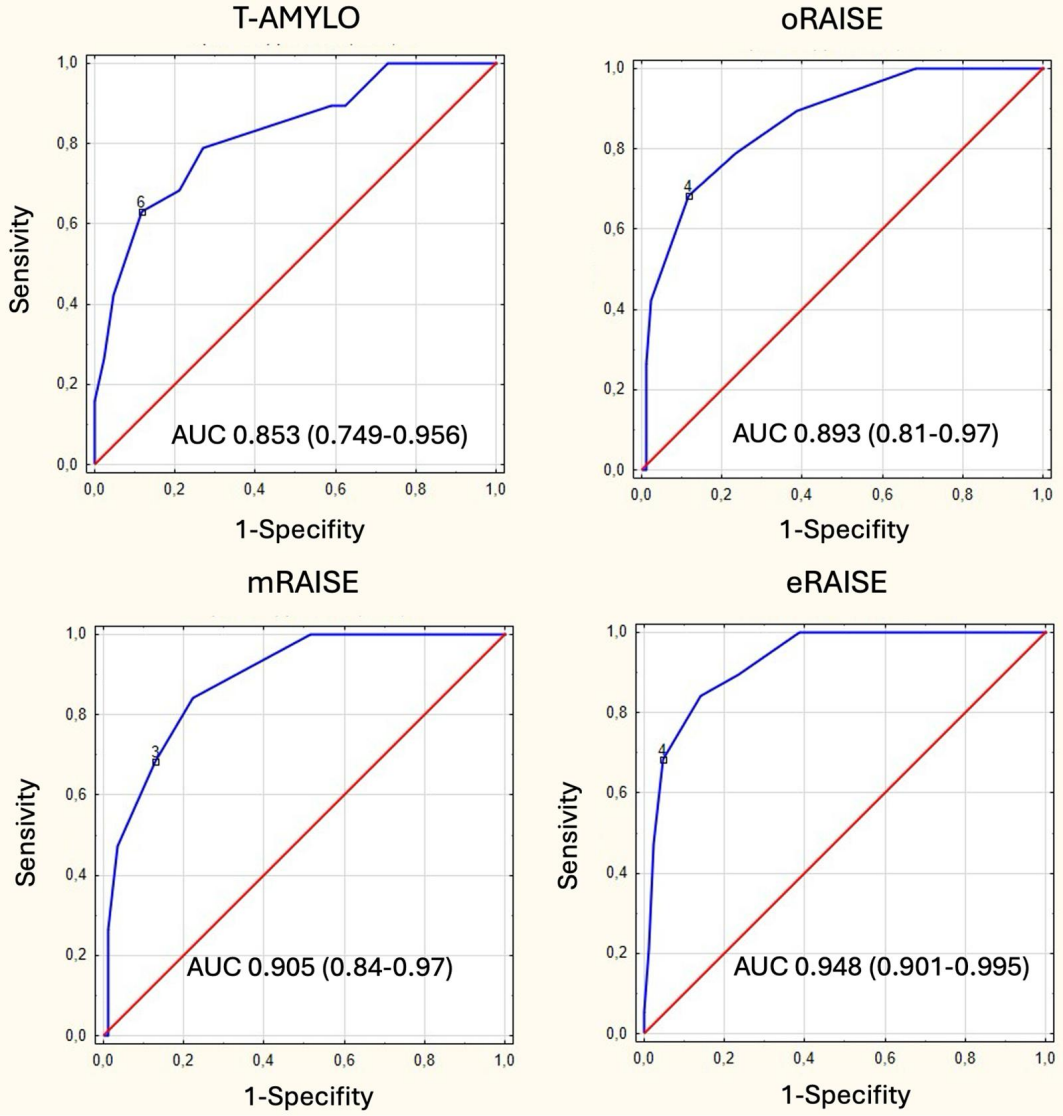
ROC curves for the presence of ATTR based on our data for available scores.

The T-AMYLO model yielded an AUC of 0.853 (95%CI 0.749–0.956). Risk stratification indicated that a score of ≥3 points denoted intermediate risk with a sensitivity of 89.5% and a specificity 43.6%. In contrast, scores ≥7 points indicated a high risk, with a sensitivity of 42.1% and a high specificity of 98.7% (Figure 1, Table 3).

**Table 3:** Diagnostic performance of the oRAISE, mRAISE, eRAISE, and T-AMYLO models for identifying transthyretin cardiac amyloidosis (ATTR-CA) in patients with severe AS.

Model	AUC [95%CI]	Sensitivity and specificity for the cut-off point adopted by the authors	Best cut-off point according to our study (sensitivity and specificity)
oRAISE	0.893 [0.81–0.97]	≥3 points (78.9%; 76.5%)	≥2 points (89.5%; 64.1%)
			For screening
			≥4 points for diagnosis
			(68.4%, 92.3%)
mRAISE	0.905 [0.84–0.97]	≥3 points (68.4%; 88.5%)	≥2 points (84.2%; 80.8%)
eRAISE	0.948 [0.901–0.995]	≥3 points (84.2%; 88.5%)	≥3 (84.2%; 88.5%)
T-AMYLO	0.853 [0.749–0.956]	Intermediate risk	≥3 points (89.5%; 43.6%)
		≥3 points (89.5%; 43.6%)	≥4 points (78.9%; 76.9%)
		High risk	
		≥7 points (42.1%; 98.7%)	

AUC, area under the curve; oRAISE, original RAISE; mRAISE, modified RAISE; eRAISE, enriched RAISE.

## 5 Discussion

Our findings are summarized as follows. First, almost one-fifth of patients with severe aortic stenosis had concomitant ATTR-CA, and one in every 20 patients had concomitant AL-CA. Second, compared to patients with AS alone, those with severe AS and ATTR-CA were older and exhibited a more severe clinical course, more advanced cardiac remodelling, and a less favourable biochemical profile. Third, patients with ATTR-CA-AS more often presented with low-gradient AS than those with AS without ATTR-CA. Fourth, the management of patients with severe AS differed between those with and without ATTR-CA, including more frequent use of TAVI and more frequent conservative treatment, reflecting worse overall clinical conditions. Finally, among the analysed ATTR scores, eRAISE showed the greatest ability to distinguish AS alone from ATTR-CA-AS, with 95% diagnostic power.

The reported prevalence of ATTR-CA among patients with severe AS varies across studies (18), ranging from 4.5% in the United States (19) to 22% in Japan (20), with a mean prevalence of 10.07%; our results align with the upper end of this spectrum in the Polish population (Supplementary Table S1). Given the substantial coexistence rate of ATTR-CA in patients with AS, several scoring systems have been developed to estimate the likelihood of amyloidosis in this population group. These tools aim to reduce the need for universal bone scintigraphy while avoiding missed diagnoses. Considering the limited availability and cost of SPECT/DPD imaging, routine testing of all patients with AS is not practical. The importance of a thorough cardiac and systemic evaluation, preferably conducted in an outpatient setting, is evident to enable earlier detection and individualized management.

Typically, ATTR-CA is associated with older age, male sex, carpal tunnel syndrome (often bilateral), characteristic ECG findings such as RBBB and low QRS voltage, and echocardiographic evidence of pronounced hypertrophy and elevated cardiac biomarkers, all of which are consistent with our observations (17, 21). After reviewing the available literature, we selected diagnostic models that were clinically relevant and feasible for routine use. We deliberately excluded models requiring complex imaging, such as CT-based or advanced echocardiographic scoring systems, as these are time-consuming, resource-intensive, and less practical in everyday care (22, 23). For clinicians managing large outpatient populations, simple and accessible tools, such as the T-AMYLO and RAISE scores, may be of much greater utility, providing rapid risk stratification to guide further diagnostic steps.

To the best of our knowledge, this is the first study to compare the diagnostic performance of these easily applicable scores in a real-world ambulatory cohort of patients with AS. Among the analysed models, eRAISE, the most advanced RAISE-derived score, performed best. It incorporates readily obtainable clinical, ECG, and echocardiographic parameters and achieves an accuracy of 95%, with both sensitivity and specificity exceeding 80% at a cut-off of ≥3 points, making it the most effective screening score in our cohort. In contrast, T-AMYLO, while less specific (<50%) with only slightly higher sensitivity (approximately 90%), proved highly reliable as a confirmatory tool: patients scoring ≥7 points had only a 5% probability of not having ATTR-CA even without DPD scintigraphy.

The RAISE family of scores is primarily designed as a high-sensitivity screening tool that is suitable for initial assessment. Previous external validations, including those by Zenses et al., have confirmed its diagnostic utility and supported the clinical relevance of specific cut-off values in clinical decision-making (16, 17). Despite its more complex calculation pathway, RAISE remains practical because it relies only on data typically available during a standard consultation. A key distinction among the four analysed scores lies in their structure and data requirements. The oRAISE score includes five domains (remodeling, age, injury, systemic, and electrical). The mRAISE streamlines the model by adjusting the parameter weighting to improve usability. The eRAISE incorporates selected echocardiographic refinements while remaining limited to readily obtainable variables, thereby ensuring its comprehensiveness and feasibility in an ambulatory setting. In contrast, T-AMYLO relies on only five parameters (age, male sex, carpal tunnel syndrome, interventricular septal thickness ≥16 mm, and low QRS voltage), which are simple to collect but less discriminative overall.

In conclusion, nearly 20% of patients with severe aortic stenosis were found to have concomitant ATTR-CA. Among the evaluated scores, the eRAISE model exhibited the highest accuracy, followed closely by the mRAISE and oRAISE models, while the T-AMYLO score provided a distinct framework for stratifying patient risk. These findings underscore the importance of utilizing appropriate scoring systems to support the diagnosis and management of cardiac amyloidosis within the context of aortic stenosis. Identifying ATTR-CA in this population is crucial for guiding appropriate management, including considering disease-specific therapies such as tafamidis, and may guide the choice of treatment for aortic stenosis. Importantly, although available data suggest that survival after aortic valve replacement (including both TAVI and surgical AVR) may be comparable in patients with AS-ATTR-CA (17, 24), many patients with ATTR-CA present with advanced age, frailty, and multiorgan failure, resulting in high (or even prohibitive) operative risk; therefore, a transcatheter strategy is the treatment of choice in this group (25). Moreover, ATTR-CA diagnosis opens up the possibility for disease-specific therapies, which, in terms of tafamidis, are associated with significantly lower all-cause and cardiovascular mortality compared to the absence of ATTR-directed therapy. The greatest benefits are observed in patients who receive both AVR and disease-modifying treatment (26).

## 6 Limitations

This was a single-centre study with a relatively small sample size, which may have impacted the generalizability of the results. However, it was designed prospectively, enrolling consecutive patients and excluding only those with contraindications for SPECT/CT. The cross-sectional nature of the study limits the ability to draw long-term prognostic conclusions about outcomes such as survival or improvements post-TAVI. The primary focus was on evaluating existing diagnostic scores using parameters that are accessible in routine clinical practice. As a referral centre for valvular interventions and cardiomyopathies, we tend to treat more severe cases, possibly introducing a referral bias towards a higher-risk patient cohort compared to those usually seen in district centres.

## Data Availability

The original contributions presented in the study are included in the article/Supplementary Material, further inquiries can be directed to the corresponding author/s.
