# Supplementary material for: Risk-score performance for detecting transthyretin cardiac amyloidosis in severe aortic stenosis: a prospective cohort study
Source: Front Cardiovasc Med. 2026 May 22;13:1773579. doi: 10.3389/fcvm.2026.1773579 (PMC13236916; doi:10.3389/fcvm.2026.1773579)
Supplement: Supplementary file 1 [file Datasheet1.docx]

Supplementary Material

# Supplementary Data

Supplementary Material should be uploaded separately on submission. Please include any supplementary data, figures and/or tables.

Supplementary material is not typeset so please ensure that all information is clearly presented, the appropriate caption is included in the file and not in the manuscript, and that the style conforms to the rest of the article.

# Supplementary Figures and Tables

## Supplementary Figures

Figure S1. Perugini grading scale of cardiac uptake in 99mTc-DPD scintigraphy.


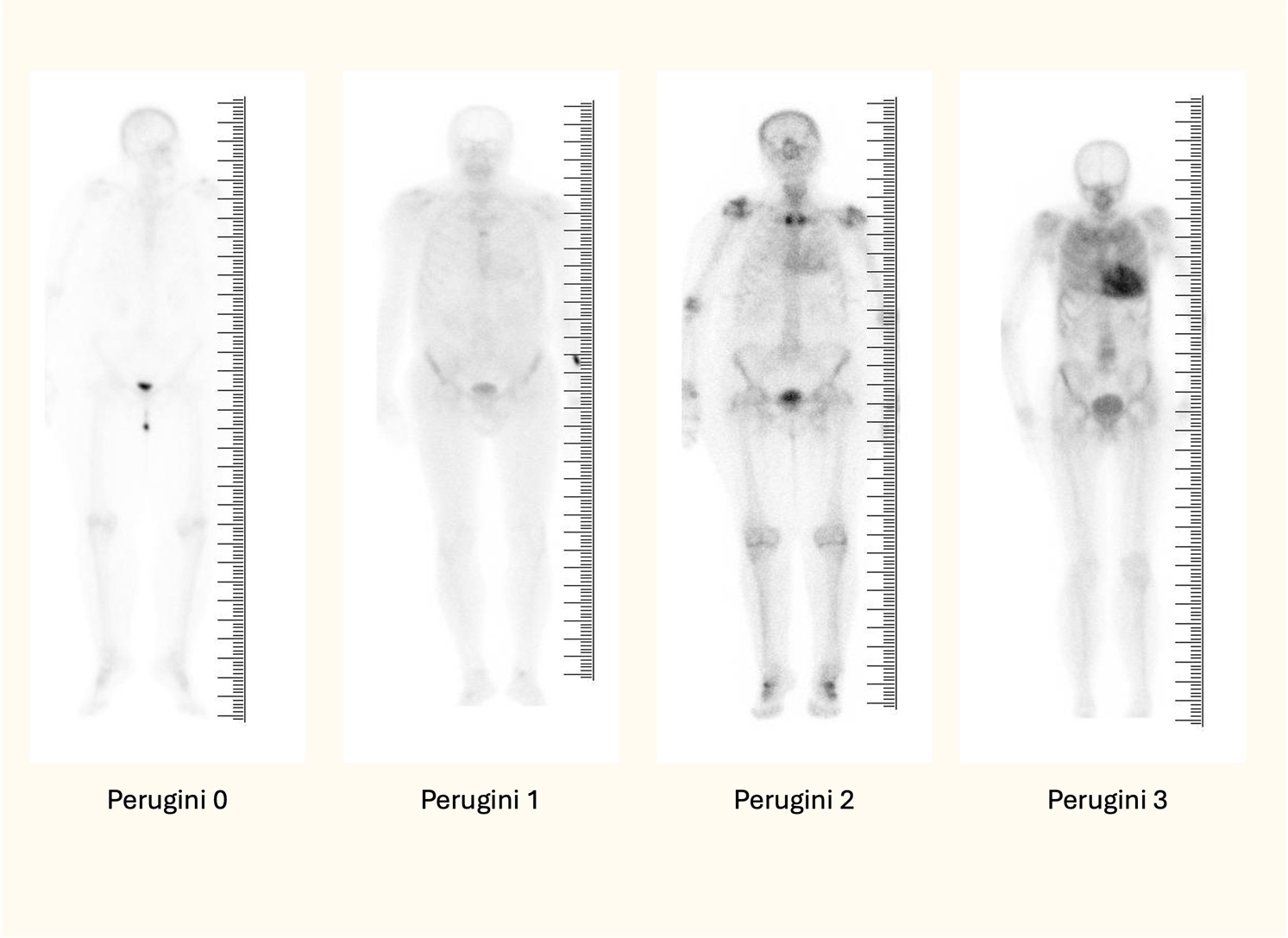


Figure S2. Diagnostic algorithm of cardiac amyloidosis based on Gilmore et al.

Figure S3. Study flowchart.

**Figure S4.** *Bland–Altman plots for echocardiographic parameters.*


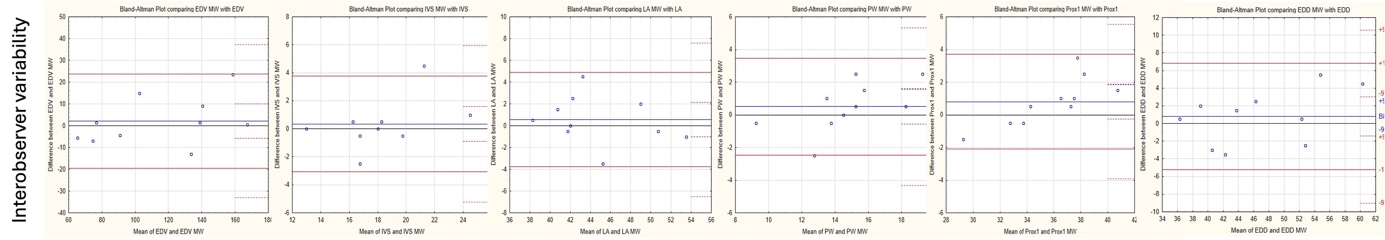


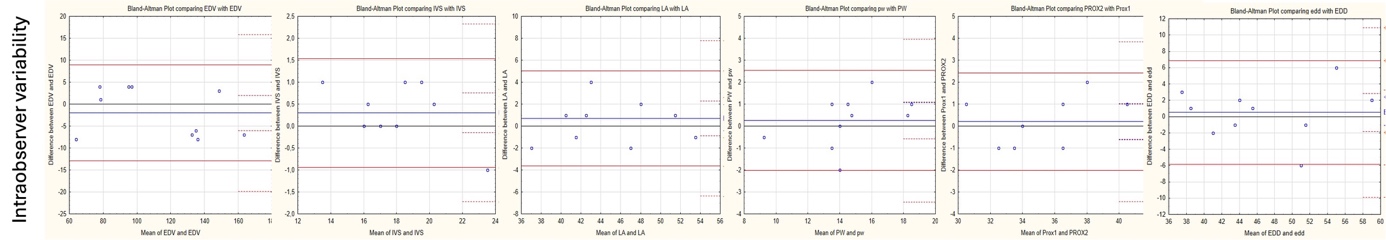


## Supplementary Tables

Table S1

| **Title/ DOI** | Number of patients with AS | % ATTR positive | Continent (country) | Predictive features |
| --- | --- | --- | --- | --- |
| **Transthyretin cardiac amyloidosis in patients with severe aortic stenosis undergoing transcatheter aortic valve replacement: experience of a single center/** [**10.23736/S2724-5683.23.06175-6**](https://doi.org/10.23736/s2724-5683.23.06175-6) | 107 | 6/13  high sensitivity (score >=2: sensitivity 93.6%, false negative rate 6.4% | Europe (Italy) | Low voltage of qrs, higher left ventricle mass index  pericardial effusion  lower voltage to mass ratio  older |
| **Canadian experience with the RAISE score to identify patients at high risk for cardiac amyloidosis in a TAVI population//10.1016/j.acvdsp.2022.10.105** | 251 | eRAISE would have triaged as high risk 44% less patients than mRAISE with similar sensitivity | North America  (Canada) | Carpal tunel or ruptured biceps tendon or lumbar stenosis; RBBB, lower voltage to mass ratio  85 years and older, elevated hsTNT without renal impairment, IVS>=18mm, LF/LG and LVEF<40% |
| **Valve disease in cardiac amyloidosis: an echocardiographic score/**[**10.1007/s10554-023-02901-2**](https://doi.org/10.1007/s10554-023-02901-2) | 423  Score odroznia AL. Od ATTR CM | AUC of 0.765  261 patients had CA, 144 ATTR-CA, 117 AL.-CA | Europe (Italy) | shortened/hidden and restricted posterior mitral valve leaflet (PMVL), thickened mitral chordae tendineae and aortic stenosis |
| **Routine 4D Cardiac CT to Identify Concomitant Transthyretin Amyloid Cardiomyopathy in Older Adults with Severe Aortic Stenosis/10.1148/radiol.230425** | 263 | 27  AUC=0,89 (081-094)  sensitivity and specificity in the detection of ATTR-CM were 96.3% (95% CI: 81.0, 99.9) and 58.9% (95% CI: 52.3, 65.2), | Europe (Switzerland) | LV GLS, Relative apical longitudinal strain, LVMassI, LA GLS |
| **Development and Validation of a Prediction Model and Score for Transthyretin Cardiac Amyloidosis Diagnosis: T-Amylo/ 10.1016/j.jcmg.2023.05.002** | 105 | 17  AUC 0,88 (0,81-0,95) | Europe (Spain) | Carpal tunnel syndrome, age>=80y, male, IVSd>=16mm, low voltage QRS |
| **Unveiling transthyretin cardiac amyloidosis and its predictors among elderly patients with severe aortic stenosis undergoing transcatheter aortic valve replacement/10.1093/eurheartj/ehx350** | 151 | 16% (24/151) | North America (USA) | Thicker IVS, higher LVMi, lower SVi, LVEF, GLS and average of lateral and septal mitral annular tissue Doppler S'; Average S' best predicted ATTR-CA in multivariable logistic regression |
| **Prevalence and Outcomes of Concomitant Aortic Stenosis and Cardiac Amyloidosis/10.1016/j.jacc.2020.11.006** | 407 | 47/407  (48 patients (11.8%; grade 1: 3.9% [n = 16]; grade 2/3: 7.9% [n = 32]). AL was diagnosed in 1 patient with grade 1) | Europe (UK, Austria) | A clinical score (RAISE) that used left ventricular remodeling (hypertrophy/diastolic dysfunction), age, injury (high-sensitivity troponin T), systemic involvement, and electrical abnormalities (right bundle branch block/low voltages) was developed to predict the presence of AS-CA (area under the curve: 0.86; 95% confidence interval: 0.78 to 0.94; p < 0.001 |
| **Light-Chain and Transthyretin Cardiac Amyloidosis in Severe Aortic Stenosis: Prevalence, Screening Possibilities, and Outcome.**  **Nitsche C, Aschauer S, Kammerlander AA, et al.**  **European Journal of Heart Failure. 2020;22(10):1852-1862. /10.1002/ejhf.1756.** | 191 | 15 (7,9%), 1 AL-CA | Europe (Vienna) | VMR as well as SVi showed good discriminative power (AUC 0.770 and 0.773, respectively), which was comparable to extracellular volume by CMR (AUC 0.756). Also, VMR and SVi were independently associated with CA by multivariate logistic regression analysis (P = 0.016 and P = 0.027, respectively  VMR – voltage mass ratio |
| **Amyloid Transthyretin Cardiomyopathy in Elderly Patients With Aortic Stenosis Undergoing Transcatheter Aortic Valve Implantation/10.1161/JAHA.123.030271** | 315 | 30 (9.5%) | Europe (Switzerland) | male sex (82.4% versus 57.7%; P=0.005), and prior carpal tunnel surgery (17.6% versus 4.3%; P=0.007) were associated with coexisting ATTR-CM, as were ECG (discordant QRS voltage to left ventricular wall thickness [42% versus 12%; P<0.001]), echocardiographic (left ventricular ejection fraction 48.8±12.8 versus 58.4±10.8; P<0.001; left ventricular mass index, 144.4±45.8 versus 117.2±34.4g/m2; P<0.001), and hemodynamic parameters (mean aortic valve gradient, 23.4±12.6 versus 35.5±16.6; P<0.001; mean pulmonary artery pressure, 29.5±9.7 versus 25.8±9.5; P=0.037). |
| **Unveiling outcomes in coexisting severe aortic stenosis and transthyretin cardiac amyloidosis./10.1002/ejhf.1974** | 204 | 27 (13%) | North America (USA) | In Cox proportional-hazards models, the presence of ATTR-CA was not associated with death. However, patients with ATTR-CA had increased rates of heart failure hospitalization at 1 year |
| **Prevalence of ATTR-CA and high-risk features to guide testing in patients referred for TAVR/10.1007/s00259-023-06374-2** | 380 | 17 (4.5%) | North America, USA |  |
| **Transthyretin cardiac amyloidosis in patients after TAVR: clinical and echocardiographic findings and long term survival/10.1002/ehf2.13667** | 88 | 11 (12.5%) | Asia  (Israel) | LVMi and pulmonary artery pressure were significantly higher, GLS and myocardial work efficiency were significantly lower in patients with ATTR-CM |
| **Concomitant Transthyretin Amyloidosis and Severe Aortic Stenosis in Elderly Indian Population: A Pilot Study/0.1016/j.jaccao.2021.08.008.** | 32 | 3 (9.4%) | Asia (India) | low myocardial contraction fraction (median [interquartile range], 28.8% [23.8% to 39.1%] vs 15.3% [9.3% to 16.1%]; P = 0.006), deceleration time (215 [144 to 236] ms vs 88 [60 to 106] ms; P = 0.009) and global longitudinal strain (-18.7% [-21.1% to -16.9%] vs -14.2% [-17.0% to -9.7%]; P = 0.030) |
| **Transthyretin amyloid cardiomyopathy in aortic stenosis patients scheduled for transcatheter aortic valve implantation/10.1002/ehf2.15258** | 171 | 8 (4.7%) | Europe (Norway) | Most ATTR-CM had low-flow low-gradient (LFLG) AS, and 25% had a history of carpal tunnel syndrome |
| **Transthyretin amyloid cardiomyopathy in severe aortic stenosis submitted to valve replacement: a multicenter study/10.1080/14796678.2024.2393031** | 91 | 11% | Europe (Portugal) | Patients with ATTR-CM were older (median age 83 vs. 77 years, p = 0.002) and had a higher frequency of carpal tunnel syndrome (100.0 vs. 2.5%, p < 0.001), mostly bilateral (n = 7, 70.0%), higher frequency of low voltage on ECG (60.0 vs. 3.8%, p < 0.001) and higher median value of NT-proBNP |
| **Utility of left atrial and ventricular strain for diagnosis of transthyretin amyloid cardiomyopathy in aortic stenosis/10.1002/ehf2.13909** | 72 | 16 (22%) | Asia (Japan) | The ^99m^ Tc-PYP scintigraphy positivity in patients with RapLSI ≥ 1.0 and the peak LSR in LA ≤ 0.47 s^-1^ was 83.3% (5/6) |
| **Multi-modality artificial intelligence-based transthyretin amyloid cardiomyopathy detection in patients with severe aortic stenosis/10.1007/s00259-024-06922-4** | 263 | 27 (10.3%) | Europe (Switzerland) | CT strain achieved the highest ROC-AUC of 0.85 (0.05) (sensitivity of 0.90 (0.11), speci-  ficity of 0.74 (0.11) |
| **Screening for Occult Transthyretin Amyloidosis in Patients with Severe Aortic Stenosis and Amyloid Red Flags/10.3390/jcm13030671** | 264 screened  85 analysed | 6 (7%) | Europe (Germany) | Syncope was more commonly reported in AS-ATTR patients (50% vs. 6%, *p* = 0.010), who also tended to have more severe hypertrophy (IVSd of 18 vs. 16 mm, *p* = 0.075). Pericardial effusion and CTS were more common in patients with dual pathology |

**Table S2.** Distribution of Perugini grades (0–3) on DPD scintigraphy in patients diagnosed with cardiac amyloidosis – transthyretin and light chain (ATTR-CA vs. AL-CA, respectively).

| Perugini grade | ATTR-CA (n=19) | AL-CA (n=5) | p-value |
| --- | --- | --- | --- |
| 0, n (%) | 0 (0%) | 2 (40%) | 0.003 |
| 1, n (%) | 0 (0%) | 1 (20%) |  |
| 2, n (%) | 3 (15.8%) | 0 (0%) |  |
| 3, n (%) | 16 (84.2%) | 2 (40%) |  |

Appendix

**Methods**

Thickness of intraventricular septum and posterior wall were registered in the parasternal long axis view, and LV mass indexed on body surface area (LVMI) was calculated as follows:

$LVM = 0.8\times\{1,04[{(LVEDD + IVSd + PWd)}^{3}-\mathrm{LVEDD}^{3}]\} + 0.6$,

and relative wall thickness:

$$RWT = (2 \times PWd)/(LVEDD)$$

where LVEDD is left ventricular end-diastolic dimension, IVSd is intraventricular septum diastolic diameter, and PWd is posterior wall diastolic diameter.

AVA was calculated according to formula:

$AVA=\frac{LVOT area\times LVOT VTI}{AV VTI}$,

where AV VTI (transvalvular pressure gradient) was calculated using continuous-wave (CW) Doppler from 5-chamber apical view (5CH), LVOT VTI (LV outflow tract VTI) – in 5CH view with pulsed-wave Doppler, and LVOT area – calculated based on LVOT diameter measured in long-axis images in maximal systole according to formula:

$$LVOT Area=\pi\times\left( \frac{LVOT diameter}{2} \right)^{2}$$

**The RAISE score** is meant for use *specifically* in patients with *severe aortic stenosis* (AS) who are being considered for TAVR (transcatheter aortic valve replacement). The RAISE score combines the following factors: each assigned a certain number of points:

| **Parameter** | **Points** |
| --- | --- |
| **Clinical** | |
| Carpal tunnel syndrome | 3 |
| Age ≥ 85 years old | 1 |
| **Electrocardiogram** | |
| Right Bundle Branch Block | 2 |
| Low voltage | 1 |
| If no Bundle Branch Block or pacemaker: Sokolow index <1.9mV | 1 |
| **Echocardiography** | |
| IVS 18mm and more | 1 |
| If sinus rhythm E/A >1.4 | 1 |
| Laboratory | |
| Hs-TnT > 20ng/L | 1 |

**Modified RAISE score (mRAISE)**

| **Parameter** | **Points** |
| --- | --- |
| **Clinical** | |
| Carpal tunnel syndrome (diagnosis or symptoms) | 3 |
| Age ≥85 years old | 1 |
| **Electrocardiogram** | |
| Complete Right Bundle Branch Block | 2 |
| Microvoltage with TTE LVH or normovoltage with severe LVH and normal BMI | 1 |
| **Echocardiography** | |
| IVS 18mm and more | 1 |
| Laboratory | |
| Positive troponin without severe renal insufficiency | 1 |

**Enhanced RAISE score (eRAISE)**

| **Parameter** | **Points** |
| --- | --- |
| **Clinical** | |
| Carpal tunnel syndrome/bicipital tendon rupture (diagnosis/symptoms) OR spinal stenosis OR other tendon rupture OR neuropathy | 3 OR 2 |
| Age ≥85 years old | 1 |
| **Electrocardiogram** | |
| Complete right bundle branch block without severe pulmonary pathology or obstructive sleep apnea | 2 |
| Microvoltage with TTE LVH or normovoltage with severe LVH and normal BMI | 1 |
| Permanent pacemaker | 1 |
| **Echocardiography** | |
| IVS ≥18mm | 1 |
| LF/LG and ejection fraction 40% and more | 1 |
| 9<IVS<12mm (Women)  10<IVS<12mm (Men)  AND RWT≥ 0.42 | -1 |
| 9<IVS<12mm (Women)  10<IVS<12mm (Men)  AND RWT≤ 0.42 | -2 |
| IVS <10mm (Women)  IVS<11mm (Men) | -3 |
| Laboratory | |
| Positive troponin without severe renal insufficiency | 1 |
| Negative troponin regardless of renal insufficiency | -3 |

**Abbr.:** BMI – Body mass index, IVS - intraventricular septum thickness in diastole, LF/LG – low flow/low gradient aortic stenosis, LVH -left ventricular hypertrophy, RWT – relative wall thickness

**T-AMYLO** includes different phenotypes of patients – HFpEF, AS, acute HF, uses a combination of readily available clinical information, ECG findings, and echocardiographic measurements to estimate a patient's risk. The factors included in the final score are:

| **Parameter** | **Points** |
| --- | --- |
| **Clinical** | |
| Carpal tunnel syndrome | 3 |
| Male sex | 3 |
| Age ≥80 years old | 1 |
| **Electrocardiogram** | |
| Low voltage | 2 |
| **Echocardiography** | |
| IVS ≥16mm | 2 |

***Reproducibility and inter-/intra-observer variability.*** *Intra- and inter-observer variability were assessed in a random subset of examinations (n=10) for right ventricular output tract (RVOT), intraventricular septum (IVS), end-diastolic diameter (EDD), posterior wall (PW), left atrium (LA) and end diastolic volume (EDV). Interobserver variability (ED vs. KG) yielded coefficients of variability of 4.12% for RVOT, 3.2% for IVS, 6.55% for EDD, 10.23% for PW, 4.93% for LA and 9.57% for EDV. Intraobserver variability (observer 2 vs observer 1) yielded coefficients of variability of 3.20% for RVOT, 3.50% for IVS, 6.95% for EDD, 7.94% for PW, 4.95% for LA and 4.94% for EDV. For Perugini grading (n=24), interobserver variability yielded a CoV of 15.32% and intraobserver variability a CoV of 10.52%. Bland–Altman plots are provided in Figure S4.*

**
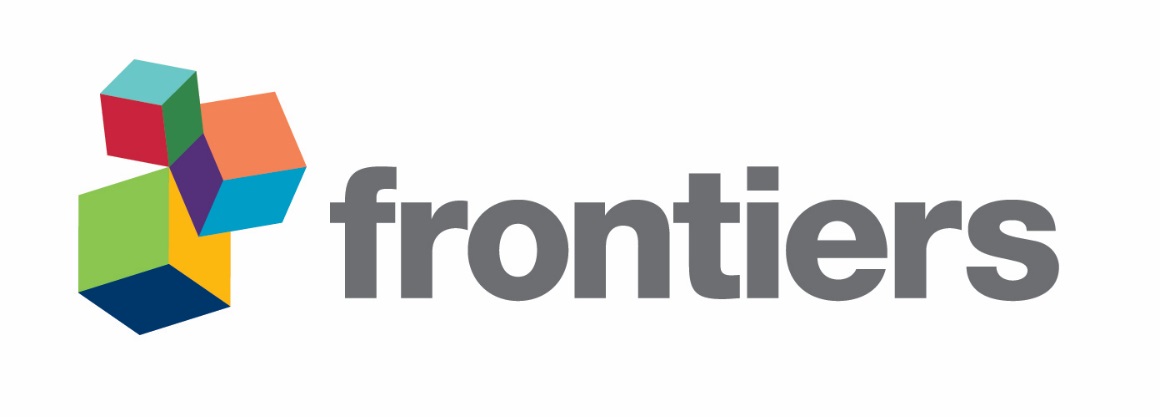
**

**Supplementary Figure 1.** The figure legends are required to have the same font as the main text, 12 point normal Times New Roman, single spaced. Please use a single paragraph for each legend and prepare the figures keeping in mind the PDF layout.
